# Supplementary material for: Cyclin-Dependent Kinase 4/6 Inhibitors and Dermatologic Adverse Events: Results from the EADV Task Force “Dermatology for Cancer Patients” International Study
Source: Cancers (Basel). 2023 Jul 18;15(14):3658. doi: 10.3390/cancers15143658 (PMC10377938; doi:10.3390/cancers15143658)
Supplement: Supplementary file 1 [file cancers-15-03658-s001.zip › cancers-2436215-supplementary.pdf]

Supplementary files

Table S2

Predictor variable	Onset 1	Onset 2	Onset 3
Age	p>0.05	Grade level 2 (p=0.018, RRR=0.93)	p>0.05
CDK	p>0.05	p>0.05	p>0.05
Pruritus	p>0.05	p>0.05	p>0.05

Table S3

Predictor variable	Onset 1	Onset 2	Onset 3
Age	p>0.05	VLL (p=0.043, RRR=1.16)	p>0.05
CDK	p>0.05	p>0.05	p>0.05
Pruritus	eczematous rash, (p=0.001, RRR=14.00), maculopapular rash (p=0.011, RRR=11.20), lichenoid, (p=0.050, RRR=11.20), vitiligo-like reaction (p=0.011, RRR=6.72).	p>0.05	p>0.05

Table S1. Naranjo algorithm

Naranjo Adverse Drug Reaction Probability Scale				
**Question**	Yes	No	Do Not Know	Score
1. Are there previous *conclusive* reports on this reaction?	+1	0	0	
2. Did the adverse event appear after the suspected drug was administered?	+2	-1	0	
3. Did the adverse reaction improve when the drug was discontinued or a *specific* antagonist was administered?	+1	0	0	
4. Did the adverse event reappear when the drug was re-administered?	+2	-1	0	
5. Are there alternative causes (other than the drug) that could on their own have caused the reaction?	-1	+2	0	
6. Did the reaction reappear when a placebo was given?	-1	+1	0	
7. Was the drug detected in blood (or other fluids) in concentrations known to be toxic?	+1	0	0	
8. Was the reaction more severe when the dose was increased or less severe when the dose was decreased?	+1	0	0	
9. Did the patient have a similar reaction to the same or similar drugs in *any* previous exposure?	+1	0	0	
10. Was the adverse event confirmed by any objective evidence?	+1	0	0	
**TOTAL SCORE:**				

Modified from: Naranjo CA et al. A method for estimating the probability of adverse drug reactions. Clin Pharmacol Ther 1981; 30: 239-245.
